# Gitelman Syndrome: A Case Report

**DOI:** 10.7759/cureus.38418

**Published:** 2023-05-02

**Authors:** João Rocha, Mariana Pacheco, Mariana Matos, Susana Ferreira, Jorge S Almeida

**Affiliations:** 1 Internal Medicine, Centro Hospitalar Universitário de São João, Porto, PRT

**Keywords:** slc12a3, metabolic alkalosis, gitelman syndrome, salt-losing tubulopathy, hypokalemia, hypomagnesemia

## Abstract

Gitelman syndrome is a rare hereditary tubulopathy characterized by hypokalemic metabolic alkalosis, hypomagnesemia, and hypocalciuria. In this case report, we describe a 21-year-old male who presented with myalgias, asthenia, general muscle weakness, and hypokalemia after receiving oral potassium supplementation for six months. Additional biochemical studies showed hypomagnesemia, metabolic alkalosis, and increased urinary potassium and magnesium excretion. Calcium urinary excretion was within the normal range, but 25-hydroxycholecalciferol levels were low. Systolic arterial hypertension was found, probably reflecting chronic hyperreninemic hyperaldosteronism. Genetic testing for *SCL12A3* mutations identified a pathogenic variant in homozygosity, which confirmed the Gitelman syndrome diagnosis. Treatment with chronic potassium and magnesium oral supplementation was started, as well as eplerenone and amiloride, with sustained correction of hypokalemia and hypomagnesemia.

## Introduction

Gitelman syndrome (GS) is a rare autosomal recessive disorder affecting the thiazide-sensitive sodium-chloride cotransporter (NCC), with an estimated incidence of one in 40,000 individuals [1]. This transporter is present in the distal convoluted tubule, and it contributes to 5-10% of renal sodium reabsorption [2]. The lack of function of NCC increases the amount of sodium present in the collecting duct, increasing the urinary excretion of potassium and hydrogen, and promoting the reabsorption of urinary calcium and excretion of magnesium. Clinically, these patients present with hypokalemia, metabolic alkalosis, hypomagnesemia, hypocalciuria, and hyperreninemic hyperaldosteronism [3]. We report a case of a young male patient with symptomatic hypokalemia diagnosed with GS, showing several biochemical findings that completely reflect the inherent pathophysiology.

## Case presentation

A 21-year-old male presented to the internal medicine department of our hospital with asthenia, myalgias, and general muscle weakness. Six months before, he had reported the same symptoms, and he was observed in a local emergency department, where severe hypokalemia was registered (2.5 mEq/L). Potassium supplementation was given, and the patient was discharged with potassium chloride tablets for five days. After completing treatment, the symptoms recurred, and he was admitted to the same hospital with severe hypokalemia (2.0 mEq/L). Serum potassium levels were corrected with parenteral therapy, and he was discharged with potassium chloride tablets (7200 mg per day) and spironolactone 25 mg id. Then, the patient was referred to our center, and he was electively admitted for further evaluation.

At admission, he reported asthenia, myalgias, and muscle weakness. He denied laxative or diuretic consumption, as well as a history of vomiting or diarrhea. His past medical history was explored, and he reported nocturnal enuresis until he was 10 years old, as well as frequent episodes of nocturia. His parents were consanguineous, and he had two healthy brothers. None of the relatives had a history of hypokalemia or related symptoms.

His physical examination was unremarkable, except for the presence of discrete signs of dehydration and sustained isolated systolic arterial hypertension grade I (systolic arterial pressure between 140-150 mmHg) [4].

Serum and urinary biochemistry findings are summarized in Table 1, Table 2, and Table 3. Although the patient was under oral potassium chloride supplementation, hypokalemia was present (3.1 mEq/L). Hypomagnesemia (1.04 mEq/L) was also identified. The patient presented normal serum ionized calcium (1.23 mmol/L) and normal parathyroid hormone levels, although low levels of 25-hydroxycholecalciferol were found. The arterial blood gas test showed metabolic alkalosis. After the suspension of potassium supplementation, a 24-hour urine sample was collected, documenting renal potassium loss (with a K+/creatinine ratio of 98.5 mmol/g of creatinine, higher than 18 mmol/g of creatinine), as well as increased urinary magnesium excretion (11.6 mmol/24 hours, with a urinary fractional excretion of 7.4%). The urinary calcium excretion was within the normal range. Serum renin and aldosterone were evaluated, and high levels of renin (214.9 U/mL, with the sample collected while standing) and aldosterone (24.3 ng/dL, with the sample collected while standing) were found, suggesting secondary stimulation of the renin-angiotensin-aldosterone axis.

**Table 1:** Relevant biochemical tests performed TSH: thyroid-stimulating hormone; PTH: parathyroid hormone

Serum biochemistry parameters	Values presented	Normal values
Haemoglobin (g/dL)	17.8	13.0-18.0
White blood cell count (/uL)	9 420	4 000 – 11 000
Platelets (/uL)	196 000	150 000 – 400 000
Urea (mg/dL)	35	10-50
Creatinine (mg/dL)	0.61	0.8-1.3
Sodium (mEq/L)	138	135-147
Potassium (mEq/L)	3.1	3.5-5.1
Chloride (mEq/L)	90	101-109
Magnesium (mEq/L)	1.04	1.55-2.05
Ionized calcium (mmol/L)	1.23	1.16-1.31
Inorganic phosphorus (mg/dL)	2.3	2.7-4.5
25-hydroxycholecalciferol (ng/mL)	16	> 30
PTH (pg/mL)	11.7	10.0-65.0
TSH (UI/mL)	1.47	0.35-4.94
Free thyroxin (ng/dL)	1.15	0.70-1.48
Renin - standing (U/mL)	214.9	5.0-55.0
Aldosterone – standing (ng/dL)	24.3	3.0-22.0

**Table 2:** Arterial blood gas test results PaCO2: arterial partial pressure of carbon dioxide; PaO2: arterial partial pressure of oxygen; HCO3: bicarbonate

Arterial blood gas test	Values presented	Normal values
pH	7.473	7.35-7.45
PaCO2 (mmHg)	43.4	35-45
PaO2 (mmHg)	85.4	80-100
HCO3^-^ (mmol/L)	31.1	22-26
Lactate (mmol/L)	2.04	< 2.00

**Table 3:** 24-hour urine sample biochemical tests

24-hour urine sample	Values presented	Normal values
Collected volume (mL)	2200	-
Sodium (mmoL/24h)	282	40-220
Potassium (mmol/24h)	183	25-125
Magnesium (mmol/24h)	11.6	6.0-10.0
Total calcium (mmol/24)	4.5	5.0-15.0
Creatinine (mg/24h)	1857	800-2000

ECG evaluation showed sinus rhythm without characteristic findings of hypokalemia. Abdominal ultrasonography was performed, and no signs of nephrocalcinosis or structural urinary tract abnormalities were found.

Considering the presence of hypokalemia due to renal losses, metabolic alkalosis, and hypomagnesemia, the diagnosis of hereditary tubulopathy was considered. After informed consent was obtained, a genetic test for GS was carried out. DNA was extracted from peripheral blood lymphocytes. Polymerase chain reaction (PCR) amplification and Sanger sequencing of exons 6 and 15 of the *SLC12A3* gene, as well as respective exon-intron boundaries, were performed. A pathogenic variant was identified in homozygosity in the splicing zone of exon 6 of the *SLC12A3* gene (c.852+1G>A), confirming the diagnosis. No pathogenic variants of *CLCNKB* were identified.

The patient was given parenteral potassium and magnesium supplementation with correction of electrolytic disturbances. Since painful gynecomastia was reported with spironolactone, he was started on eplerenone, but due to economic restraints limiting dose increase, amiloride was also associated, as were potassium chloride oral tablets. After discharge, the patient has been followed up in the internal medicine clinics of our hospital with controlled serum potassium, magnesium, and calcium levels.

## Discussion

Gitelman syndrome is a rare, heterogeneous disease typically characterized by the presence of hypokalemia, metabolic alkalosis, hyperreninemic hyperaldosteronism, hypomagnesemia, and hypocalciuria, although variable clinical presentation and severity can occur. Most patients are diagnosed during adolescence or adulthood, but neonatal presentation and diagnosis may also occur [5]. This phenotypic variability is associated not only with the *SLC12A3* mutation identified but also with the presence of other modifier genes, the coexistence of compensatory mechanisms, sex, and dietary and environmental factors [6].

Clinically, a variety of symptoms can be reported related to the biochemical abnormalities found in these patients. The most frequent complaints include cramps, muscle weakness, fatigue, and tetany [7,8]. Although thirst and salt cravings are frequent symptoms due to renal salt wasting and subsequent hypovolemia [7,8], these complaints were not reported by our patient. In fact, sustained arterial hypertension was registered in this case, and this may reflect chronic secondary hyperreninemia with juxtaglomerular cell hyperplasia due to the persistent renal salt wasting observed in GS patients [9]. Polyuria and nocturia are also frequently reported due to urinary salt and water wasting. In this patient, these symptoms started early in life, with nocturnal enuresis and nocturia episodes in adulthood. Abnormal glucose metabolism is also common in GS [10]. During outpatient follow-up, a diagnosis of type 2 diabetes mellitus was established in our patient, but the relationship with GS was unclear since other risk factors for glucose metabolism impairment were identified (namely obesity and dyslipidemia).

Biochemical findings are crucial to the diagnosis of GS, and this patient showed classical abnormalities related to this syndrome, such as hypokalemia with inappropriate renal potassium wasting, metabolic alkalosis, hypomagnesemia, and hyperreninemic hyperaldosteronism. Although the 24-hour urine sample may be more accurate, spot urine samples are usually adequate to evaluate the renal excretion of potassium, magnesium, calcium, sodium, and chloride [11]. Hypocalciuria and concurrent hypomagnesemia are strong predictors of the presence of GS, but the presence of hypocalciuria is variable, and hypomagnesemia is not always present [11]. In this case, the urinary calcium excretion was normal, and this may be related to the presence of low levels of 25-hydroxycholecalciferol and decreased renal calcium reabsorption.

Several conditions may mimic GS. Diuretics and laxative abuse, as well as surreptitious vomiting, can cause hypokalemia, and these conditions should be readily excluded with a thorough clinical history and, if necessary, a urine screen for diuretics and measurements of urinary chloride excretion [11,12]. No urine screen for diuretics or urinary chloride measurements was performed in this case since serum and urine samples were collected in a controlled inpatient setting. Primary hyperaldosteronism may also occur with arterial hypertension, hypokalemia, and metabolic alkalosis [11]. In this case, since hypertension was present, this diagnosis was ruled out by measuring serum levels of renin and aldosterone, both of which were increased, suggesting secondary hyperaldosteronism. Other hereditary tubulopathies may present with similar biochemical abnormalities to GS. Bartter syndrome type 3 is associated with mutations of the *CLCNKB* gene, which codifies a chloride channel present in the basolateral membrane of tubular cells in the thick ascending limb of the loop of Henle and in the distal convoluted tubule [13]. This syndrome may present an identical phenotype to GS, and genetic testing may be essential to differential diagnosis [14]. Epilepsy, ataxia, sensorineural hypoacusis, and tubulopathy (EAST) syndrome and heterozygous mutations in the *HNF1B* gene (associated with renal cysts and diabetes) may also mimic GS [15,16]. Gitelman syndrome-like biochemical alterations may also be found with cisplatin toxicity [17] and autoimmune disorders [18]. Since phenotypic characteristics lack specificity, genetic testing with the identification of pathogenic variants of the *SLC12A3* gene is crucial to the diagnosis of GS, and it should be offered to all patients with clinical suspicion of this disease. The genetic variant identified in our patient was previously reported as probably pathogenic, with an estimated allele frequency of 1:25000 individuals [19].

The treatment of patients with GS includes a liberal salt diet (in non-hypertensive subjects) and potassium and magnesium supplementation. Other pharmacologic treatments may be considered, such as potassium-sparing diuretics, renin-angiotensin-aldosterone system (RAAS) blockers, and non-steroidal anti-inflammatory drugs [20]. In this case, a liberal salt diet was avoided since the patient was hypertensive, and due to a symptomatic recurrence of hypokalemia while taking potassium supplements, an aldosterone receptor antagonist was initiated. Spironolactone was withdrawn because of painful gynecomastia, so the patient was given eplerenone and amiloride. No further recurrence of severe hypokalemia was registered, but during follow-up appointments, although near-normal potassium levels were observed, the patient reported slight symptoms, which suggests that, in GS, there is a lack of correlation between serum potassium levels and symptoms.

## Conclusions

This case report shows a patient with typical manifestations of GS, with hypokalemic metabolic alkalosis, and hypomagnesemia. Clinical suspicion, raised by the recognition of typical biochemical features, is the first step to establishing an accurate diagnosis, confirmed by genetic testing. Nevertheless, phenotypic diversity may be challenging, such as in the presence of arterial hypertension. Hypokalemia and hypomagnesemia symptoms and complications can be managed with chronic potassium and magnesium supplementation, as well as potassium-sparing diuretics and RAAS blockers.
